# Detailed analysis of 15q11-q14 sequence corrects errors and gaps in the public access sequence to fully reveal large segmental duplications at breakpoints for Prader-Willi, Angelman, and inv dup(15) syndromes

**DOI:** 10.1186/gb-2007-8-6-r114

**Published:** 2007-06-15

**Authors:** Andrew J Makoff, Rachel H Flomen

**Affiliations:** 1 Department of Psychological Medicine, King's College London, Institute of Psychiatry, Denmark Hill, London SE5 8AF, UK

## Abstract

**Background:**

Chromosome 15 contains many segmental duplications, including some at 15q11-q13 that appear to be responsible for the deletions that cause Prader-Willi and Angelman syndromes and for other genomic disorders. The current version of the human genome sequence is incomplete, with seven gaps in the proximal region of 15q, some of which are flanked by duplicated sequence. We have investigated this region by conducting a detailed examination of the sequenced genomic clones in the public database, focusing on clones from the RP11 library that originates from one individual.

**Results:**

Our analysis has revealed assembly errors, including contig NT_078094 being in the wrong orientation, and has enabled most of the gaps between contigs to be closed. We have constructed a map in which segmental duplications are no longer interrupted by gaps and which together reveals a complex region. There are two pairs of large direct repeats that are located in regions consistent with the two classes of deletions associated with Prader-Willi and Angelman syndromes. There are also large inverted repeats that account for the formation of the observed supernumerary marker chromosomes containing two copies of the proximal end of 15q and associated with autism spectrum disorders when involving duplications of maternal origin (inv dup[15] syndrome).

**Conclusion:**

We have produced a segmental map of 15q11-q14 that reveals several large direct and inverted repeats that are incompletely and inaccurately represented on the current human genome sequence. Some of these repeats are clearly responsible for deletions and duplications in known genomic disorders, whereas some may increase susceptibility to other disorders.

## Abstract 2

A detailed segmental map of the 15q11-q14 region of the human genome reveals two pairs of large direct repeats in regions associated with Prader-Willi and Angelman syndromes and other repeats that may increase susceptibility to other disorders.

## Background

The proximal end of chromosome 15 contains many segmental duplications and is especially susceptible to genomic rearrangements and genomic disorders (recurrent disorders that are a consequence of the genomic architecture). Among the most well studied of these are Prader-Willi syndrome (PWS) and Angelman syndrome (AS) syndromes, of which about 75% are caused by interstitial deletions in 15q11-13. Because a cluster of imprinted genes lie in the deleted region, the phenotype is dependent on the parental origin of the affected chromosome. Deletions on the paternal chromosome result in PWS, whereas deletions on the maternal chromosome cause AS [1]. These deletions occur with an approximate frequency of 1 per 10,000 live births, and they generally fall into two size classes with breakpoints (BPs) within three discrete regions (BP1 to BP3) [2]. Both classes share the same distal breakpoint (BP3), at one end of deletions that extend through the PWS/AS critical region either to BP2 (class II) or to the more proximal BP1 (class I).

Besides deletions, this region of chromosome 15 is also susceptible to duplications, triplications, and translocations. The most frequent type of duplication is due to supernumerary marker chromosomes (SMCs) [3], which are small chromosome fragments that contain two inverted copies of the proximal end of the q arm with two centromeres, p arms, and telomeres. More than 50% of all SMCs are derived from chromosome 15 and account for about one in 5,000 live births [4,5]. Many of these SMC(15) duplications (also known as inv dup[15]s) involve the same breakpoint (BP3) as in PWS/AS deletions, plus two more distal breakpoints, BP4 and BP5, that have also occasionally been implicated in PWS/AS deletions [6,7]. When they include the PWS/AS critical region and are maternally inherited, duplications are associated with a variety of phenotypes including autism, seizures, mental retardation, and dysmorphism (sometimes referred to as inv dup[15] syndrome) [8,9].

Between breakpoints BP4 and BP5 is located the gene encoding the α7 nicotinic acetylcholine receptor (*CHRNA7*), part of which is duplicated in a majority of individuals (duplication allele frequency of around 0.9 [10]). This region (15q13-q14) has been shown to be strongly linked to an endophenotype of schizophrenia, namely P50 sensory gating deficit [11], which has more recently also been shown to be a phenotype of bipolar disorder [12]. The peak lod score (5.3) is due to a marker in intron 2 of *CHRNA7*, with linkage of P50 to *CHRNA7 *also being supported by pharmacologic evidence [13]. Attempts to demonstrate linkage of this region to either schizophrenia or bipolar disorder have yielded mixed results, with one study showing linkage to bipolar disorder [14] and several studies showing only weak evidence for linkage to schizophrenia [11,15-17]. There is also evidence for association with schizophrenia and bipolar disorder [18]. Together, these findings suggest that the P50 deficit may be caused by variant(s) in the *CHRNA7 *region but, if so, that this is only one of many genetic defects that increase susceptibility to the major psychoses.

The 3' part of *CHRNA7*, including exons 5 to 10, is duplicated and this has complicated further genetic studies [19]. We previously examined the sequence relationships of these and other duplications in this region and showed that the partial duplication of *CHRNA7 *(*CHRFAM7A*) is a hybrid of *CHRN7A *and an unrelated sequence *FAM7A*, of which there are several copies [20]. Both *FAM7A *and *CHRFAM7A *are transcribed, but translation is uncertain. Using available genomic sequence data, we produced a map that showed that *CHRNA7 *and *CHRFAM7A *are in opposite orientations, suggesting that an inversion of *CHRFAM7A *might have taken place. The sequence assembly NT_010194, replacing earlier incorrect assemblies, has since confirmed the main features of our map. The sequence common to the 3' ends of both *CHRNA7 *and *CHRFAM7A *is situated at one end of two segmental duplications (duplicons) of more than 200 kilobases (kb), but the full extent of the duplicons could not be determined. This pair of duplicons was among several others and arranged in a complex fashion. The duplicon containing *CHRFAM7A *is polymorphic, due to copy number variants (CNVs), because chromosomes with one or no copies of the hybrid *CHRFAM7A *have so far been identified. We recently demonstrated an association between copy number of *CHRFAM7A *and the major psychoses, with an excess of individuals having only one copy of *CHRFAM7A *among affected patients [10]. Linkage of two different idiopathic epilepsies to the *CHRNA7 *region have also been reported [21,22].

Zody and coworkers [23] described the assembled human sequence from the entire long arm of chromosome 15 and reported nine gaps, including seven in the proximal region (15q11-q14). The three breakpoints associated with PWS/AS deletions each map to one of these gaps, all of which are adjacent to duplicated regions. In order to understand better the molecular basis for these and other rearrangements on the proximal region of chromosome 15q, we examined 15q11-q14 in detail. The human genome sequence is derived from an analysis of a vast number of sequenced clones, mainly bacterial artificial chromosome (BAC) clones. Segmental duplications present an enormous challenge because it is often difficult to distinguish between sequence alignments from different duplicons and those from different haplotypes of the same duplicon [24]. Most of the clones originate from one library (RP11), which are derived from one anonymous individual. We have focused on these RP11 clones, because it provides an opportunity to conduct a detailed analysis involving only two possible haplotypes. As a result, we were able to unravel the complicated sequence relationships between many duplicons, which has enabled us to close most of the gaps, revealing the full extent of breakpoints BP1 to BP3.

## Results

### Overview of 15q11-q14

**Figure 1:**
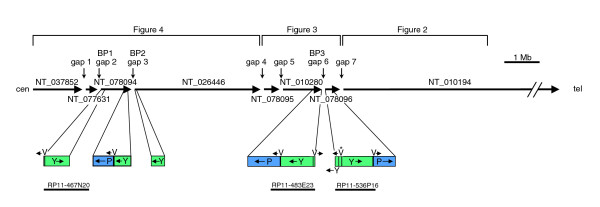
Map showing an overview of build 36 for 15q11-q14. The positions and orientations of the proximal eight contigs of 15q are shown as in build 36, with the *HERC2 *duplications (segments P, V, and Y) shown in detail. The asterisk above segment V of RP11-536P16 is to indicate that its orientation is shown as in the database. The positions of the seven gaps are shown with the approximate positions of the PWS/AS breakpoint (BP)1 to BP3. The map is divided into three parts for analysis in Figures 2, 3 and 5, as indicated. Mb, megabases.

Figure 1 shows a map of the current version of 15q11-14 in the human genomic sequence (18.2-30.8 megabases on NCBI build 36), which indicates the positions and orientations of the eight contigs that span this region. This is essentially the same as build 35, described by Zody and coworkers [23] in their analysis of 15q. Figure 1 also shows the duplicons that are adjacent to the three gaps associated with PWS/AS breakpoints BP1 to BP3, which are described in detail below.

### NT_010194

**Figure 2:**
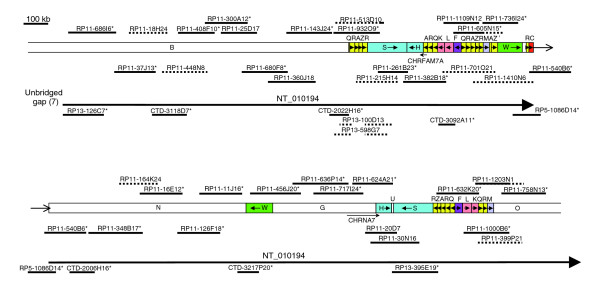
Map of 15q13-q14 at proximal end of contig NT_010194. This part of the map is an updated version of the same region that we analyzed previously [20], with some differences in segment labeling. RP11 clones representing the two possible haplotypes are arbitrarily placed either above or immediately below the segments, with the non-RP11 clones placed below the contig label. Asterisks indicate representative clones used in the contig. Solid lines indicate completely sequenced clones, and dotted lines indicate draft sequences (high throughput genomic sequences [htgs]). A solid line with a dotted line extension indicates a clone in which only a part has been completely sequenced. A gap in a clone indicates a deletion. kb, kilobases.

Since our earlier map [20], considerably more genomic DNA sequence data have become available, which we have utilized in the updated version (Figure 2). The sequence represented by the updated map is in agreement with that in the proximal (centromeric) end of contig NT_010194.

The updated map extends the proximal end of our earlier map, which terminated with an incomplete duplicated region. This duplicon has now been completed and ends inside segment Q at a junction with unique segment B (Figure 2; upper map). There is now a continuous tiling path of clones between the two ends of the map, confirming our finding that *CHRNA7 *and *CHRFAM7A *are in opposing orientation. Most of the clones originate from the RP11 library, although the clones used to define NT_010194 (shown by asterisks in Figure 2) also include some non-RP11 clones. Clones assigned to either of the two RP11 haplotypes are indicated in Figure 2 by being positioned either above or immediately below the segments, with the non-RP11 clones below the contig label. The duplicated region in the upper map, including *CHRFAM7A*, is almost completely spanned by a haplotig (a contig of clones with the same haplotype) from RP11-215H14 to RP11-540B6, confirming that it has been correctly assembled. The other duplicated region, between segments G and O (lower map), has two haplotigs (from RP11-456J20 to RP11-624A21 and from RP11-632K20 to RP11-758N13), with a gap spanned by an RP13 clone. The evidence for this RP13 clone (RP13-395E19 [GenBank: AC139426]) being located in the correct duplicon is very strong. First, it contains some of segment U, which is located between segments H and S on the duplicon in the lower map, but appears to be absent in the other duplicon. Second, the sequence of RP13-395E19 much more closely resembles that of RP11-30N16 (GenBank: AC021413) from the lower duplicon than that of RP11-261B23 (GenBank: AC135731) from the upper duplicon. In a 10 kb portion common to all three sequences there are 25 base changes and four indels when RP13-395E19 is compared with RP11-261B23, but four base changes only when it is compared with RP11-30N16 (data not shown). Conversely, there are also other RP13 clones that are closer to RP11-261B23 than to RP11-30N16. We are therefore confident that the two large regions of duplication have been correctly represented in Figure 2 and in NT_010194.

Our earlier map had a few gaps that were either spanned by clones with end sequence data only or by interpolation of missing duplicated sequence. All of these gaps have now been spanned by fully sequenced clones. There was one small error in our original map, which was caused by incorrect interpolation of missing duplicated sequence. Toward the telomeric end of our original map, we had anticipated segments QRAZR adjacent to segments M and O, because they occurred together in that order in three other places. However, at that position in the RP11 library both haplotypes have a deletion between the two R segments, leaving only QR (Figure 2, lower map). Interestingly, two RP13 clones (RP13-100D13 [GenBank: AC135991] and RP13-598G7 [GenBank: AC135994]) have paralogous deletions in QRAZR at the beginning of the first duplicon in NT_010194 (Figure 2, upper map). This deletion is clearly polymorphic, because clones representing both RP11 haplotypes have QRAZR at this position. It is possible that the deletion near the telomeric end of the map is also polymorphic and that a total of four QRAZR duplications may exist in some individuals as represented in our original map [20]. At least one of the QRAZR duplicons is therefore a CNV, but the range of copy numbers is unknown.

Another CNV in this part of 15q involves the presence or absence of the partial duplication of *CHRNA7*, the hybrid *CHRFAM7A*. We have previously shown that the homozygous null genotype is very rare, but the heteroygote occurred in 24% of psychosis patients compared to 16% of control individuals [10]. In order to define the limits of the *CHRFAM7A *deletion, we compared copy number of segments H, S and F, and H/A junction in all three genotypes using real-time polymerase chain reaction (PCR; Table 1, upper half). This showed that the deletion extends at least as far as segments S and F on either side of segments HA, where *CHRFAM7A *is found. We also amplified DNA across segmental junctions (Table 1, lower half), which showed that the deletion does not extend as far as the BQ boundary on the proximal side of *CHRFAM7A *nor as far as the MA' boundary on the distal side. This suggests that the deletion is located between the two direct repeats defined by segments QRAZR on either side of *CHRFAM7A*.

**Table 1:** Estimates for limits of duplicon containing *CHRFAM7A*

	Segment(s)	Genotype		
		
		d/d	d/n	n/n
Copy number determinations	H/A	2	1	0
	H	4	3	2
	S	4	3	2
	F	4	3	2
Segmental junction PCR	B/Q	+	+	+
	M/A'	+	+	+
	Z'/W	+	+	+
	C/N	+	+	+

Segments are as defined in Figure 2. Genotypes are defined by a d allele (containing duplicon) or n allele (lacking duplicon). Copy numbers for each segment or junction as shown. '+' indicates the presence of each segmental junction.

### Gap 7

**Figure 3:**
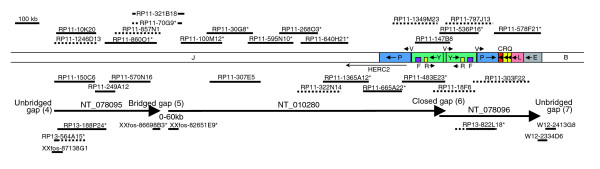
Map of contigs NT_078095, NT_010280, and NT_078096 (15q12-q13). The clones are indicated as in Figure 2. kb, kilobases.

Gap 7 separates the proximal end of NT_010194 from NT_078096. No clone in the database matches the proximal end of RP13-126C7 (GenBank: AC127522), the initial clone of NT_010194. The terminal clone in NT_078096 is RP11-578F21 (GenBank: AC055876), as shown in Figure 3, which can be extended slightly by two small non-RP11 fosmid clones: WI2-2334D6 (GenBank: AC174071) and WI2-2413G8 (GenBank: AC174069). Thereafter, no other matches could be found, so that although NT_078096 can be extended to reduce gap 7, it cannot yet be closed. This small extension of NT_078096 enables the limit of segment E, and therefore also of duplicon CRQLE, to be defined by comparison with the paralogous sequence in NT_078094.

### NT_078096

**Figure 5:**
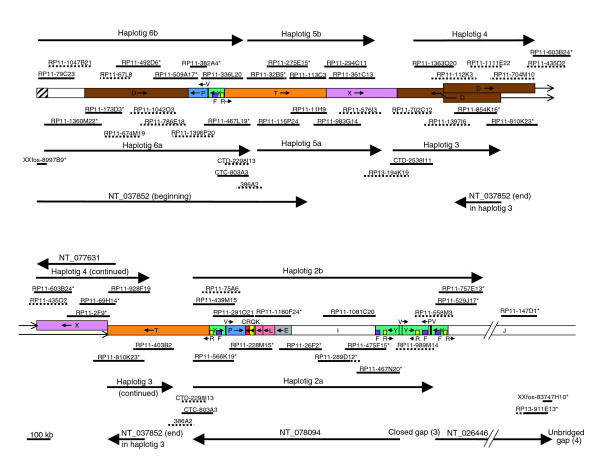
Map of contigs NT_037852, NT_077631, NT_078094, and part of NT_026446 (15q11-q12). The clones are indicated as in Figure 2. The shaded segment indicates α-satellite DNA sequence. Note that clones CTD-2298I13, CTC-803A3, and 386A2 occur twice to indicate two possible locations with respect to the RP11 sequence. kb, kilobases.

NT_078096 consists entirely of duplicated sequence. It closely resembles sequence in NT_078094, nearer to the proximal end of the chromosome. Within both of these contigs are duplicons, with smaller versions found in NT_010194. In NT_078096 are segments YVPCRQLE (Figure 3); in NT_078094 are YVPCRQKLE (Figure 5, lower map); and in NT_010194 are RQKL in two locations (Figure 2 upper and lower maps), and CR in a third location (Figure 2, upper map). Relative to both RQKL duplicons in NT_010194, all of K and some of adjacent Q are deleted in NT_078096, whereas another part of segment Q is deleted in NT_078094. By contrast, part of segment L is deleted in both NT_010194 sequences as compared with the two more proximal duplicons.

Segments R, Q, K, and L in NT_010194 have very high sequence identities with each other (>99%), but the R segment adjacent to segment C is much less similar (93%). The sequence identities between these segments in NT_078096 and NT_078094 are in the 97% to 99% range, as they are for segments Y, V, P, C, and E. Comparing segments K, Q, and R in either contig with those in NT_010194, the sequence identities are similar, but those for segments C (96%) and L (95%) are lower. Ten of the 11 R segments in these three contigs therefore have sequence identities in excess of 97%. This segment is essentially the same as the low copy number repeat (LCR15-3) described by Pujana and coworkers [25], which occurs many times elsewhere in chromosome 15 with lower sequence identity [23]. One of these is within segment Y, which has a sequence identity of 91% with the above R segments. Another sequence within segment Y has similarly moderate sequence identity (92%) with segment F. We have identified a total of seven Y segments in 15q11-q14, some of which are described below. These therefore include seven R-like and F-like segments, giving a total of 18 R segments and nine F segments for the entire 15q11-q14 region.

### Gap 6

Many gaps in the human genomic sequence are in duplicated regions and this relationship is also evident in the proximal region of 15q [23]. Five of the duplications adjacent to gaps appear to be derived from the same region (Figure 1; beginning of NT_078094, NT_026446 and NT_078096, and end of NT_078094 and NT_010280). They all include part of the *HERC2 *gene, which is located near the end of contig NT_010280 (Figure 3), from where the duplications presumably originate. Examination of the sequence at the beginning of NT_078096 revealed part of an inverted repeat (segments Y and P) on either side of a 12.6 kb sequence (segment V). There is also a 1.9 kb duplication of one end of segment V located within the inverted repeats, which is indicated in Figure 1 and elsewhere by the small segment between segments P and Y. Very similar sequence is observed on either side of gap 6, but the sequence at the end of NT_010280 contains no inverted repeat because it terminates inside segment V. As presented in the database, the two clones flanking gap 6 cannot overlap because each version of segment V appears to be in opposite orientation. However, because the first clone of NT_078096 (RP11-536P16 [GenBank: AC138749]) contains parts of both repeats, these cannot be reliably distinguished and therefore no confidence can be placed on the designated orientation for the intervening segment V in the final assembled sequence for the clone. We have previously found other examples of BAC clones containing duplicated sequence being wrongly assembled [20]. The failure of NT_010280 and NT_078096 to overlap may therefore be a consequence of misassembly.

**Figure 4:**
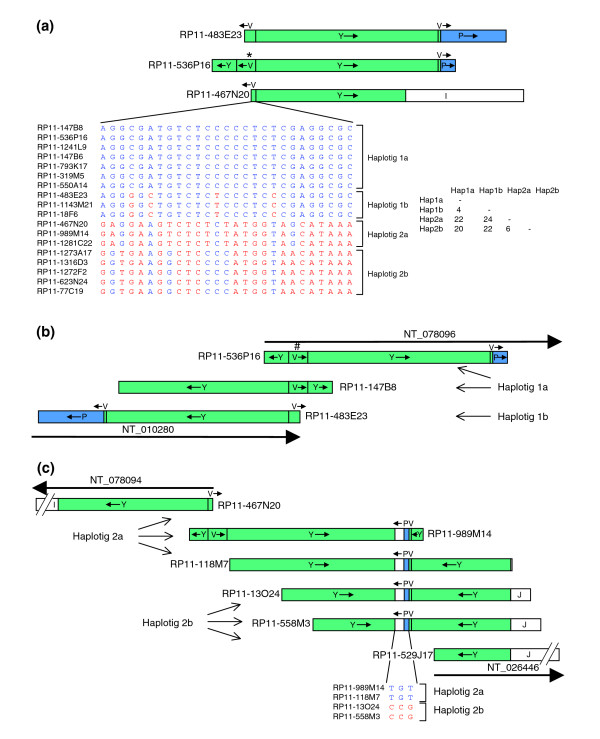
Alignment of 15q11-q13 clones in duplicons adjacent to segment V. **(a) **The three representative clones containing segment V are aligned, with single nucleotide variants in a 3,356 base pair (bp) region of segment V in all sequenced RP11 clones shown below. The asterisk above segment V indicates its orientation, as in Figure 1. The box shows the number of mismatches between each pair of haplotigs. **(b) **Corrected alignment of clones to show true relationship between ends of contigs NT_010280 and NT_078096. The hash above segment V of RP11-536P16 is to indicate that its orientation has been inverted compared with that in the database. **(c) **Alignment of clones around the segment V end of contig NT_078094, with single nucleotide variants in a 9.5 kilobase (kb) region around the small segment P shown below.

BLAST searching with segment V sequence revealed a total of 18 RP11 clones with very similar sequences (Figure 4a). Not all clones contain the entire 12.6 kb of segment V, with the 3,356 base pair (bp) region at one end of RP11-467N20 (GenBank: AC116165) at the beginning of NT_078094 representing the minimum sequence present in all 18 clones. We compared this sequence between the clones, most of which are in draft form and include ambiguous base calls designated Ns. Sequence comparisons identified many insertions and deletions, often in simple repeats, which were difficult to analyze because the repeats are prone to sequencing errors and frequently included Ns. However, we also identified 27 single base substitutions, which are not close to any Ns and are therefore likely to be real. Examination of these base changes together reveals four haplotigs, in which there are two pairs of closely related haplotigs, with four and six differences (likely to be single nucleotide polymorphisms) within haplotig pairs, as compared with 20 to 24 differences (likely to be paralogous sequence variants) between pairs (Figure 4a). For those clones in which segment V was complete, the same pattern continued throughout the segment (data not shown). This pattern strongly suggests that the first two groups of clones represent both RP11 haplotypes (haplotigs 1a and 1b) for the duplicon covered by the two adjacent ends of NT_010280 and NT_078096. The second two groups (haplotigs 2a and 2b) therefore cover the other duplicon, including the beginning of NT_078094.

The terminal clones of the two contigs flanking gap 6 (RP11-483E23 [GenBank: AC091304] and RP11-536P16 [GenBank: AC138749]) are therefore from different RP11 haplotypes. RP11-536P16 and six other RP11 clones contain sequence from haplotig 1a, including RP11-147B8 (GenBank: AC138747), where the sequence is also complete. RP11-147B8 and RP11-536P16 therefore contain overlapping sequence from the same duplicon, but they are only identical throughout segment V. None of segment Y is identical between the clones, including the uniquely represented parts, which can be reliably interpreted and which, consequently, must be derived from different duplicons. Consistent with this interpretation, uniquely represented segment Y sequence in RP11-147B8 is more similar to that of RP11-483E23 from the other haplotype of the same duplicon. Therefore, the clones overlap with relative orientations as shown in Figure 4b, demonstrating that segment V is presented in the wrong orientation in RP11-536P16. By BLAST searching for identical overlapping sequences among RP11 clones, it was possible to extend both haplotigs from NT_078096 into NT_010280, both of which therefore close gap 6 (see Figure 3).

Closing gap 6 enables the full extent of the inverted repeat to be revealed, with the two repeat segments PY extending 260 kb on the proximal side of segment V and 210 kb on the telomeric side. The size asymmetry is caused by several deletions in segment P in NT_078096 compared with NT_010280, with 96% to 97% sequence identity overall. The other more proximal P segments (in NT_078094 and NT_037852) are very similar to the segment P in NT_078096 (>99%). The Y segments exhibit a different pattern. The two paralogous sequences in the above inverted repeat (in NT_010280 and NT_078096) are very closely related with more than 99% sequence identity. The other more proximal Y segments (in NT_078094, NT_037852, and NT_026446) are slightly less closely related both to the distal pair and to each other (98% to 99%).

### Gap 5

Gap 5 is one of three gaps that do not contain adjacent duplicated sequence. The terminal clones of the flanking contigs are both small non-RP11 fosmid clones (Figure 3). Next to these are RP11-1860O1 (GenBank: AC136896) on NT_078095 and RP11-70G9 (GenBank: AC135326) on NT_010280. Although the most recent version of RP11-70G9 (GenBank: AC135326.6) has only 17,350 bp of sequence, version 5 is also complete and identical except that more sequence (41,921 bp) was deposited. This earlier version provides a perfect alignment with part of RP11-1860O1 on NT_078095, but evidently it contains a large deletion because it fails to align the intervening part of this clone (Figure 3). Another clone, RP11-321B18 (GenBank: AC107457) also spans the two contigs, with a similar but non-identical deletion. Because both clones have identical sequence to both RP11-1860O1 on NT_078095 and RP11-100M12 (GenBank: AC104002), the adjacent clone on NT_010280, it is very unlikely that they are each derived from different RP11 haplotypes. The most likely explanation is that all four RP11 clones are derived from the same haplotype, but that two clones have incurred deletions during or subsequent to cloning. Therefore, gap 5 can be bridged but, because of the presumed postcloning deletions, its exact size is unknown. Its maximum limit (approximately 60 kb) is determined by the size of the insert in RP11-70G9 before the deletion, which, judging by other RP11 clones, is unlikely to exceed 240 kb (Figure 3).

### Gap 4

Gap 4 separates the proximal end of NT_078095 from NT_026446 and lies wholly within *GABRA5*. The terminal clone of NT_026446 is the fosmid clone XXfos-83747H10 (GenBank: AC145196; see Figure 5), but the distal end does not match any other clone. The proximal end of initial clone RP13-564A15 (GenBank: AC136992) of NT_078095 matches the small fosmid clone XXfos-87138G1 (GenBank: AC145167), extending the contig slightly (Figure 3), but no further matching clones were found, and so gap 4 cannot yet be closed.

### NT_078094

As described previously, the initial clone of contig NT_078094, RP11-467N20, begins inside segment V and has sequence from haplotig 2a (Figure 4a). Two other clones, both with sequences in draft form, also have segment V from haplotig 2a. The other haplotype (haplotig 2b) is present in five clones, in which all of the sequences are only available in draft form. This region appears to have a similar sequence to that flanking gap 6, with inverted repeats on either side of segment V. The inverted repeat unit in RP11-467N20 is much shorter than that in NT_078096 and NT_010280, deviating from the other sequences before reaching the end of segment Y and therefore lacking segment P. Sequence analysis of the above clones containing haplotigs 2a and 2b showed that RP11-989M14 (GenBank: AC121153) and RP11-1281C22 (GenBank: AC136693) contained more of segment Y than RP11-467N20, plus a 9.5 kb sequence of which 3.2 kb is from segment P with the remaining sequence unique. This suggests that these two clones overlap RP11-467N20 in segment V but contain the other inverted repeat unit. BLAST searching with the 9.5 kb region from RP11-989M14 identified three other RP11 clones that also contain it, again with draft sequences available only. By using sequence alignments between these RP11 clones, it was possible to assemble all of these sequences (Figure 4c). Sequence comparisons of the 9.5 kb region identified three single base substitutions that were not near to Ns, suggesting only two haplotigs differing by three single nucleotide polymorphisms, which is consistent with a unique locus.

Two of these clones, RP11-13O24 (GenBank: AC016033) and RP11-558M3 (GenBank: AC138750), contain more unique sequence (segment J). BLAST searching with part of this sequence surprisingly identified a perfect match with RP11-529J17 (GenBank: AC100756), the initial clone of NT_026446. Further sequence comparisons confirmed that these three clones share overlapping sequence from the same RP11 haplotig 2b (data not shown). These results close gap 3 and clearly show that the beginning of NT_078094 is directly connected to the beginning of NT_026446 (Figure 4c). One of these contigs is therefore in the wrong orientation, but this cannot be NT_026446 because its other end is correctly oriented with respect to NT_078095, with *GABRA5 *spanning gap 4. NT_078094 is therefore in the wrong orientation in build 36 and in earlier versions.

We then examined the rest of NT_078094 and the two contigs proximal to it. NT_078094 consists of seven clones (shown by asterisks in Figure 5), all of which are from RP11. Sequence comparisons of the overlaps show that six clones have sequence from the same haplotype (haplotig 2a), as indicated below the segments map. The only clone used to define the contig that is from the other RP11 haplotype (haplotig 2b) is RP11-1180F24 (GenBank: AC138649), and is shown above the segments. Other RP11 clones representing most of this haplotype were also identified and show an identical arrangement of segments, supporting its correct position. Although RP11-1180F24 has part of the duplicon also found in NT_078096 and in NT_010194, in each case there are several diagnostic differences, as described earlier, making its placement in NT_078094 unambiguous. Therefore, although designated in the wrong orientation, NT_078094 represents the correct tiling path for the seven clones.

### NT_037852

The most proximal contig, namely NT_037852, comprises 11 clones, of which ten are from RP11. The first seven of these clones appear to correctly represent a tiling path (Figure 5, top left), with the initial fosmid clone (XXfos-8997B9) extending the RP11 clones by an additional 3.5 kb at the proximal end. Both RP11 haplotypes are represented (haplotigs 6a and 6b), and, when supplemented by other RP11 clones, both haplotigs are almost complete, strongly supporting the designation of that part of the contig. The proximal 43 kb of NT_037852 contains α-satellite DNA, as shown by multiple alignments within this region with a monomer sequence (for instance, L08557 from chromosome 17). This confirms the location of that end of the contig near to the centromere [26]. The next two clones of NT_037852 (RP11-32B5 [GenBank: AC068446] and RP11-275E15 [GenBank: AC060814]) share a haplotype with three other RP11 clones (Figure 5, haplotig 5b), with the other RP11 haplotype being plausibly represented by four other clones (Figure 5, haplotig 5a), although there is an alternative possibility (see below). The final two clones of NT_037852 (RP11-810K23 [GenBank: AC037471] and RP11-854K16 [GenBank: AC126335]) are part of a five-clone haplotig (Figure 5, haplotig 3).

### NT_077631

The above haplotigs show that each of the three parts of contig NT_037852 is internally consistent. In order to understand the likely relationship between them, we also must consider the adjacent contig NT_077631. This comprises three RP11 clones RP11-69H14 (GenBank: AC134980), RP11-2F9 (GenBank: AC010760), and RP11-603B24 (GenBank: AC025884), which are clearly all from the same haplotype and therefore correctly assembled. This haplotype can be extended in both directions by other RP11 clones to create a very long haplotig of nine clones (Figure 5, haplotig 4). At one end of haplotig 4 are two truncated D segments, oriented in a head to head manner. The D/D junction is unlikely to be a cloning artefact because it is present in two independent clones from the same haplotype (RP11-1363O20 and RP11-112K3). At the other end of haplotig 4 are segments T and X. Along with haplotigs 5a and 5b, this is the third RP11 haplotig to include these segments. Either of haplotigs 5a or 5b could be allelic with haplotig 4, but, as discussed below, this is unlikely.

### The proximal end of 15q

**Figure 6:**
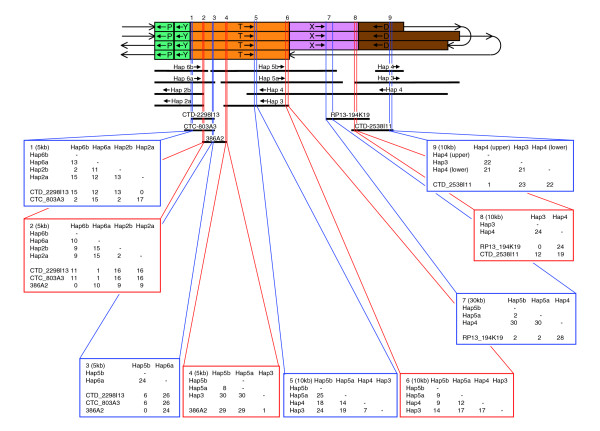
Analysis of symmetrical region near the centromeric end of 15q to identify its likeliest arrangement in RP11. The region between the most proximal segments P ordered as in Figure 5 is indicated by the four rows of segments at the top. The first row, continuing to the third row, represents the upper RP11 haplotigs in Figure 5 and the second row, continuing to the fourth row, represents the lower haplotigs. The RP11 haplotigs are shown below the segments with the non-RP11 clones shown further below. Nine slices of 5 to 30 kilobases (kb), shown by alternating red or blue lines, were investigated, with each box showing the number of single nucleotide mismatches between each pair of RP11 haplotigs and non-RP11 clones in the slice.

All RP11 clones that map centromeric to NT_026446 belong to a total of eight haplotigs from duplicated regions (Figure 5). Haplotigs 2a and 2b (NT_078094) are clearly allelic, as are haplotigs 6a and 6b (NT_037852, beginning). In order to determine whether haplotigs 5a and 5b are also allelic, they were compared in 5 or 10 kb slices with the homologous region in haplotig 4 (Figure 6). In segment T there was moderate to high variation, with variation between haplotigs 5a and 5b being no more similar to each other than either was to haplotig 4 (Figure 6, slices 4 to 6). By contrast, in segment X variation was much lower, so that three adjacent 10 kb slices were required in order to obtain a sufficient number of base substitutions for meaningful comparison. In this 30 kb region, there were only two base changes between haplotigs 5a and 5b, as compared with 30 base changes between either with haplotig 4 (Figure 6, slice 7). This pattern continued in a region of at least 100 kb of segment X, which contained only seven base changes between haplotigs 5a and 5b, both of which differed from haplotig 4 by 94 base changes (data not shown). They also differed from haplotig 4 by two large indels: two versus three perfect 29 bp repeats, and eight versus ten imperfect 37 bp repeats. These observations strongly suggest that haplotigs 5a and 5b are allelic, with haplotig 4 being part of another duplicon.

**Figure 7:**
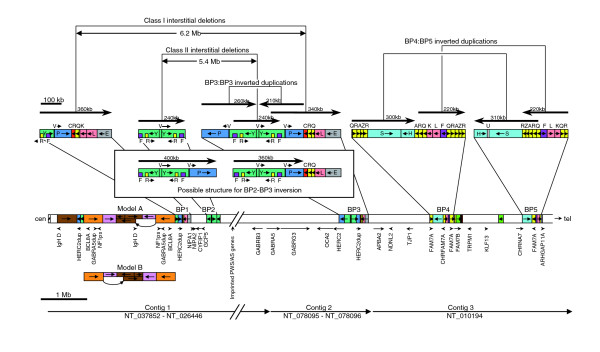
Map showing positions of segmental duplications of 15q11-14 in the RP11 individual. The main part of the map shows the segmental duplications as in Figures 2, 3 and 5, with the approximate positions of genes, duplications (dup), and pseudogenes (ps) shown underneath and the positions of the three remaining contigs at the bottom. Note that most of the imprinted region in the Prader-Willi/Angelman syndrome critical region is not included. The alternative structure (model B) near the centromere is shown underneath. The duplicated regions in each of the five breakpoint regions (BP1 to BP5) are shown in more detail above the map and include the probable structure for those individuals with a BP2:BP3 inversion. The positions of the major direct and inverted repeats are shown above the detail with arrows in an arbitrary direction.

Of the eight RP11 haplotigs at the proximal end of 15q, three pairs are therefore allelic, leaving haplotigs 3 and 4 apparently nonallelic. It is possible that RP11 sequence exists that is allelic with haplotigs 3 and 4, for which clones have not been isolated. However, because nine RP11 clones all contain sequence from haplotig 4 and five more are from haplotig 3, this seems unlikely. It is more likely that the RP11 individual is heterozygous for a complex CNV and that haplotigs 3 and 4 represent the two alternative alleles in such a region of segmental variation. The arrangement as shown in Figure 5 (model A) represents one way to assemble the haplotigs described for this region under this assumption. There is an equally parsimonious alternative assembly (model B), with haplotigs 5a/5b and 3/4 inverted (Figure 7). By exchanging haplotig pairs, both models also have minor alternatives that leave the arrangement of segments unaffected. RP11-32B5 in haplotig 5b and RP11-467L19 in haplotig 6a overlap (Figure 5, as in NT_037852) and exhibit a very high degree of variation, for example 24 base substitutions in a 5 kb slice of this overlap (Figure 6, slice 3). Because haplotigs 5b and 6a clearly do not represent the same haplotype, haplotigs 5b and 5a cannot be exchanged in model A. However, the overlap between haplotigs 5b and 6a could be due to an allelic overlap between different RP11 chromosomes (as in model A) or a nonallelic duplication (as in model B), and therefore - with no other sequenced RP11 clones covering this region - cannot discriminate between models A and B.

Non-RP11 clones cover some gaps between the allelic haplotig pairs and were examined in order to provide evidence to support the proximal end of the proposed map. Two such clones cover the gap between haplotigs 5a/5b and 3/4. One end of RP13-194K19 overlaps RP11-702C12 of haplotig 3 (Figure 5), with no base substitutions in a 10 kb region within the overlap (Figure 6, slice 8). Its other end (Figure 5) overlaps both RP11-576I3 (haplotig 5a) and RP11-361C13 (haplotig 5b), with only two base substitutions with either being in a 30 kb region (Figure 6, slice 7). This suggests that the RP13 individual contains a haplotype similar to the RP11 haplotigs on either side of the gap, and supports the placement of haplotig 3 adjacent to haplotigs 5a/b. Clone CTD-2538I11 overlaps RP11-1363O20 (Figure 5, upper, haplotig 4) at one end with one base substitution in a 10 kb region (Figure 6, slice 9) and RP13-194K19 at the other end with 12 base substitutions in 10 kb (Figure 6, slice 8). This is consistent with the placement of the segment D end of haplotig 4 adjacent to haplotigs 5a/5b. These observations support haplotigs 5a/5b being adjacent to 3/4, as in both models A and B, assuming no major gene conversion in segments X and D.

The two remaining gaps in the proximal region both occur in segment T and are paralogous: between the central four haplotigs (5a, 5b, 3, and 4) and haplotigs 6a/6b on one side and haplotigs 2a/2b on the other (Figures 5 and 6). To investigate the likely orientation of the central four haplotigs in order to discriminate between models A and B, we analyzed alignments with three non-RP11 clones that cover these two gaps. However, the results of this analysis were unable to discriminate between the models because they suggested that these clones were derived from haplotypes in segment T that showed similarities to both duplicons of that region in RP11. At one end, clone 386A2 is very similar to haplotig 3 (Figure 6, slice 4), with one substitution in 5 kb, whereas the middle of the clone (slice 3) exhibits strong similarity to haplotig 5b (no base substitutions in 5 kb). Similarly, clone CTD-2298I13 exhibits strong similarity to haplotig 2a at one end, but also strong similarity to haplotig 6a in the middle of the clone (Figure 6, slices 1 and 2). One end of the third non-RP11 clone, CTC-803A3, is similar to both haplotigs 2b and 6b, which are very similar to each other (Figure 6, slice 1). Comparisons between haplotigs 2a, 2b, 6a, and 6b over segment T (Figure 6, slices 1 and 2) and segments P and Y (data not shown) sometimes exhibited greater similarity within a haplotig pair than between them (for instance, slice 2), but not always (for example, slice 1). Overall, there was no greater similarity within haplotigs (data not shown). This pattern of homogenization of the two inverted duplicons is often a signature of gene conversion events [24]. These observations suggest that nonallelic homologous recombination (NAHR) has been taking place between both duplicons containing segments P, Y and T, suggesting that this region may exist in both orientations in different individuals. Although it is not possible to discriminate between models A and B in the RP11 individual, it is possible that both models for at least one of the RP11 haplotypes exist in the general population.

## Discussion

We believe that the map presented here (detailed in Figures 2, 3 and 5, and summarized in Figure 7) is the most accurate representation to date of the duplication structure of 15q11-q14 for the individual whose DNA was used in the RP11 library, and is a significant improvement on build 36 sequence in this region. Most of the seven gaps have now been closed, leaving only two clear gaps (gaps 4 and 7) plus some uncertainty near the proximal end. This means that the region is defined by just three contigs: contig 1, NT_037852 to NT_026446; contig 2, NT_078095 to NT_078096; and contig 3, proximal end of NT_010194. As the proximal end of NT_037852 has more than 40 kb of α-satellite DNA, thus reaching the pericentromeric region, the position and orientation of contig 1 are unambiguous. The other end of contig 1 (distal end of NT_026446) terminates within *GABRA5 *and includes exons 1 to 6. As exons 7 to 11 of *GABRA5 *are within NT_078095 at the proximal end of contig 2, its position and orientation are also unambiguous. The orientation of the final contig 3 is unambiguous because it is wholly within NT_010194, which is a very large contig of 54 megabases spanning 15q13-q24, and genetic and cytogenetic evidence demonstrates that the end adjacent to NT_078096 is clearly 15q13.

Within contig 1, we found that NT_078094 is presented in the database in the wrong orientation. Our evidence is very strong because we have found tiling paths from both ends of NT_078094 to each adjacent contig (NT_026446 and NT_037852). Four genes have been identified on the nonduplicated central part of NT_078094 [27], in the following order: *GCP5*, *CYFIP1*, *NIPA2*, *NIPA1*. By using PCR on a panel of yeast artificial chromosomes, this group showed that the order was cen-*NIPA1*-*GCP5*-tel, agreeing with the order shown in Figure 7 and confirming that NT_078094 is in the wrong orientation on build 36 and earlier versions. In contig 2, we identified an assembly error in a clone (RP11-536P16, AC091304) located at the beginning of NT_078096, which resulted in the segment V located between two inverted repeats being presented in the wrong orientation. This prevented the overlap between NT_078096 and NT_010280 from being recognized. As a result of correcting these two orientation errors, we were able to identify two large direct repeats and one large inverted repeat, the significance of which is discussed below.

Also within contig 1 is a region that we have demonstrated is likely to be heterozygous for complex CNVs in the RP11 individual. This region contains four genes/pseudogenes (IgH *D *region, *NF1 *pseudogene, *GABRA5 *duplication, and *BCL8A*) that have been shown to be present in different copy numbers [28,29]. The IgH *D *region is located at one end of segment D and is present in two copies in the lower haplotig, but because it is absent from both of the truncated versions of segment D it is present as only one copy in the upper haplotig. The other three genes/pseudogenes are located on segment T, which in RP11 has a copy number of two on both chromosomes. Comparative genomic hybridization and representational oligonucleotide microarray analysis studies also suggest the presence of CNVs in the pericentromeric region of 15q [30-34]. In a recent comparative genomic hybridization (CGH) study, Redon and colleagues [33] investigated copy number variation in the human genome in 270 control individuals from the HapMap collection. They found evidence for CNVs throughout 15q11-q14, which were most frequent in a region extending from the centromere to the beginning of segment J. In a similar study conducted in the same sample, Sharp and coworkers [34] also found a very high frequency of CNVs in this region, with the greatest variation occurring in regions corresponding to segments D, T, and X, which contained CNVs in 51%, 49%, and 40% of the sample, respectively. Together, these results demonstrate that there are multiple versions of the pericentromeric region within different human populations, with variable numbers of segments D, T, and X.

On either side of this variable region in both chromosomes of RP11 are two inverted duplicons containing segments P, Y, and T. When we compared clones from either duplicon, we found evidence for homogenization of variants between them, suggesting that significant gene conversion has occurred [24]. This may be due to intrachromosomal NAHR between inverted duplicons, predicting that where crossovers have occurred the region between them should exist in both orientations. There may also have been recombinations between these duplicons on SMC(15)s and chromosome 15, as discussed below, which could also account for some of the observed homogenization.

An important consequence of closing the gaps associated with segmental duplications is that, for the first time, it is possible to use reliable sequence data to analyze the five breakpoints that are responsible for the known genomic disorders on 15q11-14. The breakpoints have been mapped to specific BAC clones in several studies [7,32,35,36], which we utilized to position them on the map in Figure 7. The most frequent type of interstitial deletion found in PWS/AS patients occurs between BP3 and either BP1 or BP2. Inspection of these regions in Figure 7, reveals two pairs of large direct repeats that can readily explain such frequent deletions by NAHR between each repeat unit. For class II deletions (BP2/BP3), the direct repeats are each 240 kb in length, whereas for class I deletions (BP1/BP3) the direct repeats are larger, being 340 kb and 360 kb, but with several large gaps between homology units (data not shown). Studies of several PWS/AS patients have suggested that class II deletions are more common (approximately 60%), which appears to be inconsistent with this interpretation because the direct repeats involved in generating class I deletions are larger. This may reflect the greater overall identity between the 240 kb repeats compared with the larger direct repeat pair. However, of more likely relevance is the observation that an inversion between BP2 and BP3 is disproportionately frequently seen in one chromosome of mothers of AS patients with class II deletions (four out of eight), as compared with 9% in the general population [37]. As shown in the inset in Figure 7, the likely structure of a chromosome with this type of inversion has a much larger pair of direct repeats (360/400 kb) available for NAHR. These large repeats appear to explain the apparently high susceptibility for class II deletions in chromosomes with this inversion. If the data in the report by Gimelli and coworkers [37] are representative of class II deletions, then, among chromosomes that are noninverted at BP2/BP3, class I deletions may in fact be more common. This is consistent with observations elsewhere showing a correlation between size of duplication and rate of NAHR [38].

Other larger deletions have also been reported, but these appear to be much rarer (for example, patients with BP2/BP4 deletions) [32,36]. These can be explained by the two regions within segment Y that exhibit 91% to 92% sequence identity with segments F and R, thereby providing several small low homology direct repeats between BP2 and BP4 (one F segment on each BP; two R segments on BP2 with five R segments on BP4). However, there are direct repeats (segments RQKL) on BP1 and BP4 that are longer (120 kb) and with a much greater sequence identity, so it would be surprising if BP2/BP4 deletions were more common than BP1/BP4 deletions, which to our knowledge have not yet been unambiguously identified.

Direct repeats can produce deletions by NAHR, which may be interchromosomal, interchromatid, or intrachromatid [24]. The first two mechanisms should generate interstitial duplications as frequently as interstitial deletions, whereas the third should be specific for deletions only. Interstitial duplications involving BP1/BP3 and BP2/BP3 are observed [7,36,39,40], but they appear to be much less common than the equivalent deletions. Only if an intrachromatid mechanism accounted for a significant proportion of NAHR events would interstitial duplications be expected to be less common. The mechanisms of a few PWS deletions have been investigated, and interchromosomal recombinations were identified in five cases out of seven [41], with the remaining two being due to either of the two intrachromosomal mechanisms. It is therefore unlikely that an intrachromatid mechanism can explain the lower reported incidence of interstitial duplications compared with deletions. It is probable that the imbalance between deletions and duplications results from an ascertainment bias caused by the less severe phenotype in this type of duplication, especially in paternal duplications [39].

The most commonly observed duplications in 15q are the dicentric SMC(15)s. For example, Wang and coworkers [7] analyzed 35 patients with known duplications on chromosome 15 by CGH, and showed that only four were due to interstitial duplications (BP1:BP3). The other 31 were all due to SMC(15)s. However, as with comparisons with deletions, the lower reported incidence of interstitial duplications compared with SMC(15) duplications may also be subject to ascertainment bias. Known duplications of both types that include the PWS/AS critical region are almost always maternal in origin [6,35,39], whereas duplications excluding that region show no parental origin bias [5,9,41]. Paternal duplications involving the PWS/AS critical region do occur, but they have a milder phenotype extending into the normal range [5,39,42]. Equivalent SMC(15)s have two extra copies of the PWS/AS critical region, so that a total of three copies are maternally inherited compared with two maternal copies with interstitial duplications. Consequently, because of increased gene dosage, the phenotypes of patients with SMC(15) duplications are more severe [8].

In the 31 SMC(15)s described above [7], 18 had a recombination event between BP4 and BP5, whereas the remaining 13 were smaller with BP3:BP3 recombinations. Other, apparently rarer SMC(15)s were also reported among other patient samples analyzed in the same study: two at BP5:BP5, one at BP2:BP2, and two tricentric SMC(15)s. Locke and coworkers [32] also reported a BP2:BP2 and tricentric and monocentric SMC(15)s. Roberts and colleagues [35] reported findings similar to those of the study conducted by Wang and colleagues [7], in which 46 SMC(15)s were analyzed using a combination of fluorescent *in situ *hybridization and microsatellite mapping; 32 of the 46 exhibited two distinct breakpoints close to BP4 and BP5, and 14 out of 46 had two very closely located breakpoints near to BP3. Two smaller studies using either CGH [36] and fluorescent *in situ *hybridization [6] yielded similar findings. Together, these studies suggest that BP4:BP5 recombination events cause the most common type of SMC(15), at least among clinically significant cases, with BP3:BP3 recombinations also a common cause.

There are three pairs of large inverted repeats in 15q11-q14 (Figure 7): two pairs on BP4/BP5 and one pair on BP3, which appear to explain the most common types of SMC(15). Together, the inverted repeats on BP4/BP5 cover around 500 kb, whereas the pair on BP3 covers around 200 kb, but with more gaps between homology units and lower sequence identity in segment P (data not shown). The larger target size of the inverted repeats on BP4/BP5 and greater sequence identity presumably accounts for it being more common. The BP5 region contains a small pair of inverted repeats of segments QR at approximately 40 kb. Recombination at these repeats to produce a BP5:BP5 SMC(15) would involve the PWS/AS critical region, as for the BP4:BP5 and BP3:BP3 types of SMC(15). The severity of the phenotype is likely to be similar, and therefore the lower incidence of such SMC(15)s no doubt reflects the smaller size of the repeats. The BP2 region contains a larger pair of inverted repeats (around 100 kb), but SMC(15)s generated from recombination here would not include the PWS/AS critical region, and so they are probably under-reported.

Apart from SMC(15)s generated by BP2:BP2 recombinations, those involving more proximal recombinations also lack the PWS/AS critical region. These include those likely to be generated by the inverted duplicons containing segments P, Y, and T discussed above, and by inverted repeats in the variable proximal region (Figure 7). Because the inverted repeats with segments P, Y, and T (sometimes extending to segments X and D) are even larger (≥580 kb) than those involved in generating the common clinically significant SMC(15)s, they may be formed more frequently. There is some evidence for the existence of such small SMC(15)s [42], but they have not been well characterized. Their apparent low frequency may well be due to their probable low clinical significance. Consequently, it is likely that some SMC(15)s such as these occur in the general population.

As discussed above, the observed homogenization of the two proximal inverted duplicons containing segments P, Y, and T may be due to recombinations between SMC(15)s and chromosome 15. Because the most proximal regions of 15q are likely to be over-represented in SMC(15)s, recombinations involving proximal regions between SMC(15)s and chromosome 15 may also be over-represented. Such recombinations can generate inversions, insertions, deletions, and other rearrangements, and they may explain at least some of the high variation observed in this region of 15q. There may also be a lower but significant frequency in the general population of paternally derived larger SMC(15)s containing 15q proximal sequence, at least as distal as BP5. Other duplicated regions, for instance in the BP4 and BP5 regions close to *CHRFAM7A *and *CHRNA7 *(Figures 2 and 7), are clearly vulnerable to genomic changes mediated by NAHR within and between chromosome(s). Some of these rearrangements may also be mediated by recombinations with larger (probably paternally derived) SMC(15)s.

The polymorphism for the presence or absence of *CHRFAM7A *that we found to be associated with psychosis [10] could theoretically be caused by BP3/BP4 deletions involving interrupted direct repeats defined by segments RQL. However, we have shown that such a deletion cannot account for this polymorphism, because segment F is also deleted and the segment B/Q boundary is intact. Although a deletion between the two direct repeats QRAZR within the BP4 region is far more likely, there is a major problem with either explanation. As discussed earlier, if this is a deletion produced by NAHR occurring in several independent events, then a duplication involving the same two homologous recombination sites should occur as frequently. However, a single deletion event followed by subsequent accumulation of the *CHRFAM7A *region imposes no such constraint. We have analysed more than 1,000 samples from schizophrenia, bipolar, epilepsy, and control white Caucasian patients and have found no example of *CHRFAM7A *duplication [10]. Hong and coworkers [43] presented data suggesting that three out of 212 Chinese bipolar patients or control individuals might have a *CHRFAM7A *duplication, but this proportion is very much lower than their estimated null *CHRFAM7A *frequency. The much higher incidence of no copies of *CHRFAM7A *compared with two copies is unlikely to be due to ascertainment bias. First, the *CHRFAM7A *null allele is observed in many unselected controls. Second, it is unlikely that *CHRFAM7A *duplication would be associated with a deleterious biologic effect, because known duplications that include the CHRFAM7A region (for instance, BP4:BP5) are well characterized and, where paternally inherited, have a mild phenotype. It is therefore more likely that the null *CHRFAM7A *allele is not a deletion at all, but results from persistence of an ancestral genomic structure.

Segmental duplications are nonrandomly distributed throughout the genome, with clusters on several chromosomes [38]. Many have been implicated in genomic disorders and may also be involved in complex genetic disorders and common traits [44]. We have mapped and characterized the cluster of segmental duplications toward the proximal end of 15q in one individual. Many are associated with known rearrangements in this part of the chromosome, and are likely to be causative for at least some of these disorders. Some rearrangements are comparatively rare, but have been studied extensively because of the severe phenotypes they cause. Other rearrangements appear to be much more common, such as the BP2/BP3 inversion and the probable persistence of a pre-*CHRFAM7A *structure that predates the partial duplication of *CHRNA7*. Their phenotypes appear to be normal, although the former confers an increased risk for PWS/AS in the next generation, and there is some evidence that the latter may predispose to the major psychoses. There are likely to be many other rearrangements in this region, including one close to the centromere for which the RP11 individual appears to be heterozygous. Some of these are likely to be common. The consequences of potentially so many large genomic variants in this region of 15q may be that it is an important region for complex genetic disorders and common traits, as well as for the more deleterious phenotypes already identified. It is therefore essential that this region is sequenced in many individuals in order to define the range of common genomic variants in the human genome.

## Conclusion

We have produced a segmental map of 15q11-q14 that reveals the full extent of several large direct and inverted repeats in one individual that are incompletely and inaccurately represented on the current human genome sequence. Among these repeats are direct repeats that are responsible for the deletions that cause PWS and AS, and inverted repeats responsible for the inverted duplications that, when maternal in origin, cause genomic disorders with phenotypes that include autism. The q11-q14 region of chromosome 15 is highly unstable and is likely to be continually generating many other rearrangements, some of which may be risk factors for human diseases, including psychoses, that map to this region. Our re-evaluation of this region provides a more accurate framework to investigate these potential changes.

## Materials and methods

### Alignment of sequences of clones from public access database

Matches between clones were identified using BLASTN [45], as described previously [20]. We facilitated the analysis of alignments from the BLAST 2 SEQUENCES procedure by downloading the data into Microsoft Excel to generate a list of co-ordinates of all strong alignments for each pair of sequences. Our Excel file created for this purpose is available on request.

Having identified matching regions between two clones, we needed to distinguish between alignments that were due to overlaps of identical sequence from the same chromosome (when comparing RP11 clones), due to allelic overlaps from different chromosomes, and due to nonallelic duplications. This task was complicated by the possibility of sequencing errors and sequencing misassemblies within each clone. The latter was particularly prevalent where there were duplications within a clone. Our approach was to compare mismatches between different alignments in relationship to the context. In order to have a reasonable chance of discriminating between the three causes of alignment specified above, an overlap above a minimum size was necessary. We attempted to include end sequences from BAC clones in this analysis, but because of their small size and generally higher sequence error rate they were uninformative. Our analyses suggested that an overlap size of well in excess of 1 kb was required.

### Identification of RP11 haplotigs in duplicated regions

Each segmental duplication is represented in the RP11 library by up to two sets of allelic variants from each RP11 chromosome and differ from each other by sets of nonallelic paralogous sequence variants. Thus, for *n *duplications, there will be up to 2*n *tiling paths of clones each representing a different RP11 haplotype, which we have referred to as a haplotig. In order to identify different haplotigs representing a duplicon in the RP11 library, we performed a BLASTN alignment with a reference RP11 sequence (usually a 5 kb or 10 kb fragment). We analyzed the results by manipulating the data in an Excel file (available on request) that generates the coordinates on the reference RP11 sequence of each mismatch and identifying each base change involved.

### Copy number determinations

Copy number of *CHRFAM7A *(H/A) was determined with a Taqman assay, as described previously [10]. Copy number was also determined, using forward and reverse primers, and probes for segments H (TGAGTTTTCCACATGTACAGAACCA, GTCGGCTCCCAACTTCGT, and CTTTGGACACGGCCTCC, respectively), S (ACGTGAGTTTGTTCAAGCAAGTC, CTTCTTCCCAGCATGTCACAGAT, and CCGCCTCCACAAGTT) and F (TGAAGTGTGGGTCATTTCCTAAGC, GGCAGACACAGCTGGGATAG, and CTACAGCCATGAGCTACTG).

### PCRs across segmental boundaries

PCRs across boundaries between segments B/Q, M/A', Z'/W, and C/N were at 30 cycles of 94°C/1 min, t°C/1 min, and 72°C/3 min with primers and annealing temperature (t) as follows: B/Q (TGGAGCCAGTCCGAGATAGG, GCTTCACCACCACGACTGG, t = 63°C), M/A' (CAAACTGTAATGTGGCATCC, CAATGGTGCCTATCCCTGCC, t = 56°C), Z'/W (CTGATACATCACCAACTTGG, GAGACCAGCCTGATCAAGG, t = 62°C), and C/N (TGCTTGGGAATTTGATATGG, TGTGAATATTCCCATACAG, t = 56°C). PCR products were resolved on 2% agarose gels.

## Additional data files

The following additional data are available with the online version of this paper. Additional data file 1 contains the complete list of clones (with accession numbers) and the sequences used in the analysis, cross-referenced to the figures in which they appear.
